# The Time Course of Face Representations during Perception and Working Memory Maintenance

**DOI:** 10.1093/texcom/tgaa093

**Published:** 2020-12-15

**Authors:** Gi-Yeul Bae

**Affiliations:** Department of Psychology, Arizona State University, Tempe, AZ 85287, USA

**Keywords:** ERP decoding, face decoding, face memory, face perception, temporal dynamics of faces

## Abstract

Successful social communication requires accurate perception and maintenance of invariant (face identity) and variant (facial expression) aspects of faces. While numerous studies investigated how face identity and expression information is extracted from faces during perception, less is known about the temporal aspects of the face information during perception and working memory (WM) maintenance. To investigate how face identity and expression information evolve over time, I recorded electroencephalography (EEG) while participants were performing a face WM task where they remembered a face image and reported either the identity or the expression of the face image after a short delay. Using multivariate event-related potential (ERP) decoding analyses, I found that the two types of information exhibited dissociable temporal dynamics: Although face identity was decoded better than facial expression during perception, facial expression was decoded better than face identity during WM maintenance. Follow-up analyses suggested that this temporal dissociation was driven by differential maintenance mechanisms: Face identity information was maintained in a more “activity-silent” manner compared to facial expression information, presumably because invariant face information does not need to be actively tracked in the task. Together, these results provide important insights into the temporal evolution of face information during perception and WM maintenance.

## Introduction

Human faces contain multidimensional information that is critical for social interaction. Faces contain changeable information that varies depending on attentional and emotional states (e.g., gaze direction and facial expression). They contain invariant information that is preserved despite variable states (e.g., face identity). While a classic model for face perception proposes independent and parallel processing of variant and invariant aspects of face information (Bruce and Young 1986), more recent models propose that the two types of information are processed interactively (Haxby et al. 2000; Ganel and Goshen-Gottstein 2002; Atkinson et al. 2005). Studies also found that the face processing is more dynamic such that the processing of face identity and facial expression is influenced by various factors such as the task demand and individuals’ experiences (Yankouskaya et al. 2014).

While much research on face processing has focused on how we perceive faces, it is also important to understand how the perceived faces are maintained in working memory. For example, it is necessary to know whether the person that we are talking to now is the same person that we were talking to prior to a brief interruption, and a rapid memory for expressions may be important for monitoring how someone’s emotions change over the course of a conversation. Neuroimaging studies have identified neuroanatomical networks that are involved in working memory for face information (Druzgal and D’Esposito 2003; Neta and Whalen 2011), but little is known about the millisecond-level temporal dynamics with which face information evolve over time from perception to working memory.

The present study investigated the time course of invariant (i.e., face identity) and variant (i.e., facial expression) face information during perception and working memory. Traditional univariate ERP methods have been widely used to study face processing with high temporal precision (Itier 2004; Kappenman and Luck 2011), but these methods do not reveal *which* identity or *which* expression is being perceived or remembered. Instead, the present study used a multivariate ERP decoding approach (Grootswagers et al. 2017) that has been used to track the time course of specific visual information in perception and working memory (Bae and Luck 2018, 2019a). Prior research has used an EEG/magnetoencephalography (MEG) decoding approach to assess the temporal dynamics of face processing (Nemrodov et al. 2016, 2018; Dobs et al. 2019; Smith and Smith 2019) but these studies were mostly on the perception period and focused on determining the earliest time points where face information becomes available.

**Figure 1:**
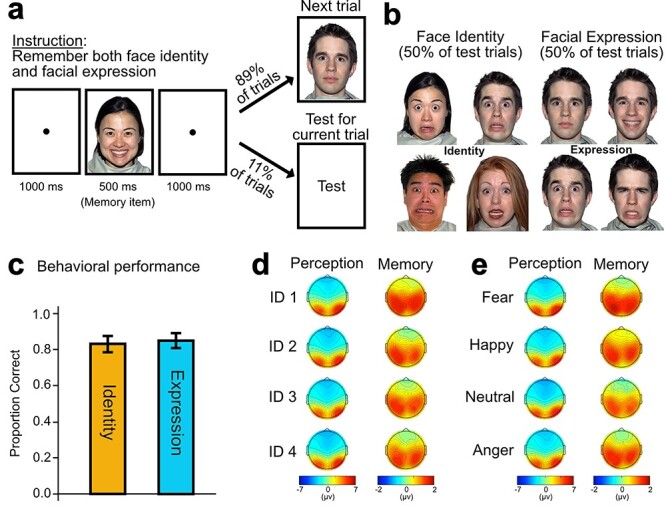
Task procedure, behavioral performance, and ERP topography. a. On each trial, participants saw a face image and remembered both the face identity (ID) and facial expression. Images shown here are not scaled to actual size. This was typically followed after a delay by the next trial. However, on a random subset of the trials, memory was tested for the face ID or facial expression from that trial. b. Two types of test trials. For the face ID test trials (indicated by “Identity” in the middle of the display), 4 face images with different face IDs but the same facial expression (except for the facial expression of the original memory item) were presented, and participants reported which of the 4 face images was the same ID as the memory item. For facial expression test trials (indicated by “Expression” in the middle of the display), 4 face images with different facial expressions of the same face ID (except for the face ID of the original memory item) were presented and participants reported which of the 4 face images was the same facial expression as the memory item. I show examples of the NimStim face images due to the restriction placed on the stimulus set (Tottenham et al., 2009). c. Behavioral performance for the ID and Expression tests (n = 22). The difference between them was not statistically significant. Error bars indicate ±1 SEM. d,e. Topography of ERP activity for each of 4 face IDs (d, collapsed across facial expressions) or facial expressions (e, collapsed across face IDs), averaged across the participants and time points during the perception (0–500 ms) and the working memory interval (500–1500 ms).

The present study used a face working memory task in which participants were required to perceive the identity (4 different identities) and emotional expression (fear, happy, neutral, anger) of a given face image during a 500-ms stimulus presentation and maintain this information across a 1000-ms delay period (Fig. 1a). Participants were then tested unpredictably on either the identity or the expression. Because the to-be-reported dimension was not known until the end of the trial (Fig. 1b), participants had to extract both types of information on every trial. The EEG was recorded while participants performed the task, and a multivariate decoding analysis was conducted to track specific face identity and facial expression information during the perception and working memory maintenance^1^.

The decoding analyses revealed a temporal dissociation between face identity and facial expression: Although the decoding of face identity was stronger than facial expression during the perception period, the decoding of facial expression was stronger than face identity during working memory maintenance period. Subsequent analysis suggested that the weaker face identity decoding during working memory maintenance does not mean that the face identity information was not stored in working memory. Instead, it suggested that face identity information might be stored in a more “activity-silent” manner (Stokes 2015; Wolff et al. 2017). These results demonstrate dissociable temporal dynamics of face identity and facial expression information during perception and working memory maintenance, providing new insights onto the model of face processing (Bruce and Young 1986; Haxby et al. 2000).

## Materials and Methods

### Participants

A total of 22 college students (14 female, 8 male) between the ages of 18 and 30 with normal or corrected-to-normal visual acuity participated for monetary compensation ($12/h). The sample size was determined prior to the data collection based on previous EEG decoding studies (Foster et al. 2016; Nemrodov et al. 2016; Bae and Luck 2018, 2019a). The study was approved by the UC Davis Institutional Review Board and the Arizona State University Institutional Review Board.

### Stimuli & Apparatus

A total of 16 face images from the NimStim facial expressions data set (Tottenham et al. 2009) (4 identities: 27 M, 36 M, 06F, and 07F; 4 expressions: fear, happy, neutral, and anger) were used. These 16 face images were chosen based on the similarities in skin color and hairstyle, and to match the number of male and female faces. The task was generated in Matlab (The Mathworks, Inc.) using PsychToolbox (Brainard 1997; Pelli 1997) and was presented on an LCD monitor (Dell U2412M) with a gray background (31.2 cd/m2) at a viewing distance of 100 cm. A black fixation dot was continuously present in the center of the display, and participants were instructed to maintain fixation on this dot except during the response period.

### Experimental Design

Procedure of the task is illustrated in Figure 1a. Each trial started with a 1000-ms presentation of the central fixation dot followed by a 500-ms presentation of a face image (5.1° × 3.7°). Identity and facial expression were randomly selected from the 16 face images. During the stimulus presentation period, participants were asked to remember both face identity and facial expression of the face image. After another 1000-ms memory interval, participants were presented with a new face image for the next trial (89% of the trials). This new face image was always in different face identity and facial expression from the previous-trial face image. For the remaining 11% of the trials, participants were provided with a 2 × 2 array of 4 face images (each image, 3.3° × 2.3°) for memory test (Fig. 1b). During the test, participants reported either face identity (50% of the test) or facial expression (50% of the test) but not both. Which face dimension should be reported was indicated by “Identity” or “Expression” presented in the middle of the display. To prevent the repetition of memory item being presented in the test display, task-irrelevant face dimension was always different from the memory item. For example, when face identity 1 expressing happy was a memory item for an identity test, images in the test display were 4 different face identities expressing one of the remaining 3 facial expressions (e.g., fear). Likewise, when face identity 1 expressing happy was a memory item for a facial expression test, images in the test display were 4 different facial expressions with one of the remaining face identities (e.g., face identity 2). The test images remained on the screen until participants pressed one of 4 buttons (corresponding to the 2 × 2 location of the test images) on a gamepad for a report. A white screen with the central fixation dot followed for 1000 ms and a new face image was presented for the next trial. Note that face memory was tested for 11% of the trials for the sake of efficiency. However, participants had to remember both face identity and facial expression every trial because when and which face dimension would be tested on a given trial were unknown.

Each participant completed a total of 720 trials plus 16 practice trials. Among the 720 main experiment trials, face memory was tested 80 times (5 trials for each of the 16 face images, in random order). I excluded trials right after the test trials from the EEG analysis because memory reports could influence EEG signals associated with the perception of the subsequent face image. Excluding the posttest trials produced 640 trials (40 trials for each of the 16 face images, in random order) for the main decoding analysis. Participants took a short break after every 80 trials. The experiment lasted about an hour.

### E‌EG Recording & Preprocessing

The continuous EEG was recorded using a Brain Products actiCHamp recording system (Brain Products GmbH). Recordings were obtained from a broad set of 59 scalp sites (FP1/2, AFz/3/4/7/8, Fz/1/2/3/4/5/6/7/8, FCz/1/2/3/4/5/6, Cz/1/2/3/4/5/6, T7/8, CPz/1/2/3/4/5/6, TP7/8, Pz/1/2/3/4/5/6/7/9/10, POz/3/4/7/8, Oz/1/2). Electrodes on the left and right mastoids were recorded and served as reference sites. The horizontal electrooculogram (EOG) was recorded from electrodes placed lateral to the external canthi and was used to detect horizontal eye movements. The vertical EOG was recorded from an electrode placed below the right eye and was used to detect eyeblinks and vertical eye movements. Electrode impedances were maintained below 50 KΩ. All signals were recorded single-ended and then referenced offline. The EEG was filtered online with a cascaded integrator-comb antialiasing filter (half-power cutoff at 130 Hz) and digitized at 500 Hz. A photosensor was used to measure the temporal delay between the onset of the stimulus on the computer monitor and the event code recording in the EEG data set. On average, there was a 26-ms delay of the stimulus onset from the event code. This delay was compensated by shifting the event code in the data set backward by 26 ms.

Signal processing and analysis was performed in Matlab using EEGLAB Toolbox (Delorme and Makeig 2004) and ERPLAB Toolbox (Lopez-Calderon and Luck 2014). The scalp EEG was referenced offline to the average of the left and right mastoids. A bipolar horizontal EOG derivation was computed as the difference between the two horizontal EOG electrodes, and a vertical EOG derivation was computed as the difference between Fp2 and the electrode below the right eye. All the signals were band-pass filtered (non-causal Butterworth impulse response function, half-amplitude cutoffs at 0.1 and 80 Hz, 12 dB/oct roll-off) and resampled at 250 Hz. Portions of EEG containing large muscle artifacts or extreme voltage offsets (identified by visual inspection) were removed. Independent component analysis (ICA) was then performed on the scalp EEG for each subject to identify and remove components that were associated with blinks (Jung et al. 2000) and eye movements (Drisdelle et al. 2017). The ICA-corrected EEG data were segmented for each trial from −500 to +1500 ms relative to the onset of the stimulus.

### E‌EG Decoding Analysis

I attempted to decode both face identity and facial expression based on the scalp distribution of the phase-locked ERP voltages (Grootswagers et al. 2017). The decoding procedure for face identity and facial expression was identical except for the way the data were organized. For the sake of brevity, I describe the procedure for face identity decoding here. To decode face identity, the trials were organized in terms of face identity (collapsed across facial expressions). This created 160 trials for each face identity (i.e., 40 trials per face identity × 4 facial expressions). The raw EEG for each trial was low-pass filtered at 6 Hz using the EEGLAB eegfilt() routine. This was determined a priori based on previous studies (Bae and Luck 2018, 2019a, 2019b) to ensure that the decoding is not influenced by alpha-band activity (8–12 Hz), which is a major source of trial-to-trial variability (see Supplementary Material for additional decoding analysis with more modest low-pass filtering and decoding analyses with ERPs from different frequency bands). This process produced a 4-dimensional data matrix for each participant, with dimensions of time (500 time points; one data point for every 4 ms for the duration of 2000 ms), face identity (4 identities), trials (160 individual trials for each face identity), and electrode site (the 59 scalp sites).

I used the combination of a support vector machine (SVM) and error-correcting output codes (ECOC) (Dietterich and Bakiri 1995) to classify the face identity on the basis of the spatial distribution of the ERP signal over the 59 scalp electrodes. The ECOC model was implemented through the Matlab fitcecoc() function. The data were decoded independently for each of the 500 time points (every 4 ms) from −500 ms to +1496 ms (relative to the stimulus onset). However, the statistical analysis for decoding accuracy focused on temporally contiguous clusters of time points (rather than focusing on each time point) and the continuous nature of EEG data was taken into account for the statistical testing (see below for statistical analysis of decoding accuracy).

I used a 3-fold cross-validation with an averaging procedure (Grootswagers et al. 2017). 3-fold was chosen to maximize the number of trials within a group so that signal-to-noise ratio can be maximized by averaging (Isik et al. 2014). The data for a given face identity were divided into 3 equal sized groups of trials (3 groups of 53 trials for each of the 4 face identities). One random trial for each of the 4 face identities was excluded because 160 is not evenly divisible by 3. The trials for a given face identity in each group were averaged together, producing a scalp distribution of ERP signals for a time point being analyzed (a matrix of 3 groups × 4 face identities × 59 electrodes). Two groups of datasets were submitted to the ECOC model with known face identity labels to train the 4 SVMs. Each SVM learned to perform a binary classification that separated one of the 4 face identities from the other 3 face identities (i.e., one-vs-rest coding design). The remaining group of datasets were used for testing which was done with the Matlab predict() function. The output of this function provides one predicted face identity for each of the 4 face identities in the testing dataset. Decoding accuracy was then computed by comparing the true face identity labels of the test data set with the predicted labels for the data set. This procedure was repeated 3 times, once with each group of data serving as the testing dataset. To minimize idiosyncrasies associated with the assignment of trials to groups and to increase reliability of the decoding, I iterated the entire procedure 10 times with new random assignments of trials to the 3 groups (see Supplementary Material for the effect of the iteration procedure). The number of iteration used here was determined a priori on the basis of previous studies (Bae and Luck 2018, 2019b, 2019a). After completing all the iterations of the cross-validation procedure, decoding accuracy was collapsed across the 4 face identities, across the 3 cross-validations, and across the 10 iterations, producing average decoding accuracy for a given time point based on 120 decoding attempts. After this procedure was applied to each time point, the averaged decoding accuracy values were smoothed across time points to minimize noise using a 5-point moving window (equivalent to a time window of ±8 ms).

The procedure for the decoding of facial expression was identical to the decoding of face identity except that the data were organized in terms of facial expression (collapsed across face identity).

Because the main goal of the present study was investigating the temporal dynamics of face identity and facial expression representations, the analysis focused on the independent decoding of the two face dimensions. However, I report the decoding of the combination of face identity and facial expression in the Supplementary Material.

### Statistical Analysis of Decoding Accuracy

If the scalp distribution of ERPs contains information about face identity, then the decoding accuracy should be higher than chance level (1/4). To compare decoding accuracy to chance at each time point while controlling for multiple comparisons, I took a nonparametric cluster-based permutation approach (Bae and Luck 2019c) that is analogous to the cluster-based mass univariate analysis commonly used in EEG research (Maris and Oostenveld 2007; Groppe et al. 2011). This method is appropriate here because decoding accuracy may not be normally distributed. In addition, it provides an intelligent correction for multiple comparisons and takes noise autocorrelation in the EEG data set into account.

To perform cluster-based permutation test, I first tested whether the decoding accuracy at each time point after the onset of the stimulus was greater than chance (1/4) using one-sample *t*-tests. One-tailed tests are appropriate here because the SVM approach could not produce meaningful below-chance decoding. After the independent *t*-test for each time point, I found clusters of contiguous time points for which the single-point *t-*tests were significant (*P* < 0.05), and the *t* scores within each cluster were then summed together to produce a cluster-level *t* mass. Each cluster-level *t* mass was then compared against a null distribution for the cluster-level *t* mass that was determined via permutation analysis.

To perform the permutation analysis, I randomly permuted the true labels for the test data prior to computing decoding accuracy rather than permuting the labels for the data and then going through the training–testing procedure (Bae and Luck 2019c). This enabled faster reconstruction of the null distribution. By permuting the true label for the test data set, the decoding accuracy is necessarily at the chance level. Crucially, I used the same permuted label for each time point in a given EEG epoch, to account for temporal autocorrelation in the data during the construction of the empirical null distribution. After computing decoding accuracy with permuted labels, the decoding accuracy was smoothed with a 5-point running average filter (the same procedure applied to the actual decoding accuracy). I then found clusters of contiguous time points using the method described above. If there were no significant clusters of time points, then the cluster mass was set to zero for that permutation trial. If there were more than one cluster of significant time points, I took the mass of the largest cluster.

I repeated the whole permutation process 1000 times with randomly permuted true face identity label and constructed the empirical null distribution of the cluster-level *t* mass, resulting in the resolution of *P* = 10^−3^. I then computed the *P* value corresponding to each cluster in the actual data set by examining where each observed *t* mass fell within the null distribution. The *P* value for a given cluster was set based on the nearest percentiles of the null distribution. If the obtained cluster-level *t* mass was larger than the maximum of permuted cluster-level *t* mass, then I reported *P* < 0.001. I rejected the null hypothesis and concluded that the decoding was above chance for any observed cluster-level *t* mass that was in the top 95% of the null distribution (alpha = 0.05).

To statistically compare decoding accuracies between face identity and facial expression, I used the same cluster-based permutation approach, but with two differences. First, I used two-tailed *t-*tests rather than one-tailed *t*-tests because one face dimension could be decoded better or worse than the other dimension. Second, the null distribution of the cluster-level *t* mass was constructed by randomly swapping labels for the two conditions across participants 1000 times. I computed the *P* value corresponding to each cluster in the actual data set by examining where each observed *t* mass fell within the null distribution, and rejected the null hypothesis if the observed *t* mass fell within the top or bottom 2.5% of values from the null distribution (two-tailed, alpha = 0.05).

### Temporal Generalization

To test the temporal evolution of brain activation patterns for a given stimulus class, I conducted cross-time decoding analysis in which a classifier was trained on one time point and tested on all the time points (Grootswagers et al. 2017). To conduct this analysis, data within a 100-ms time window (e.g., [−500, −400] ms, [−400, −300] ms and so on.) were averaged prior to the training and testing. The time-averaging process produced 20 data points for the entire epoch for a given trial ([−500, 1500] ms). Decoding was done with the all the possible combination of training and testing time points (20 × 20). All other aspects of the decoding analysis were identical to the main decoding analysis.

### Cross-Dimension Decoding Analyses

The main decoding analysis for a given face dimension was done independently of the other face dimension. Although the other face dimension (e.g., facial expression for face identity decoding) was randomly assigned for the training and testing data set, it is possible that trials for a stimulus image were present in the training and testing data set, influencing the decoding. To rule out this possibility and to test the independency in a more stringent way, I conducted cross-dimension decoding analyses, similar to leave-one-exemplar-out cross validation (Grootswagers et al. 2017) and independent exemplar cross validation (Carlson et al. 2014). To decode face identity, I trained the face identity classifier using trials with 3 out of 4 facial expressions (e.g., fear, neutral, and anger) and tested the classifier for the trials with the remaining facial expression (e.g., happy). Likewise, to decode facial expression, I trained the facial expression classifier using trials with 3 out of 4 face identities (e.g., identities 1–3) and tested the classifier for the trials with the remaining face identity (e.g., identity 4). Because a decoding-irrelevant face dimension was not repeated between the training and testing data sets, above chance decoding from this analysis provides stronger support for the independency between the two face dimensions (e.g., face identity information plays no role in facial expression decoding). This cross-dimension decoding analysis was repeated 4 times until each of the 4 levels of decoding-irrelevant face dimension served as test data set (4-fold-cross validation). Because the data for training and testing were controlled rather than randomly assigned as in the main decoding analysis, iterations with random assignment could not be applied. Consequently, decoding results were less reliable than the main decoding analysis. All other aspects of the cross-dimension decoding procedure were identical to the main decoding procedure, including statistical testing.

### Decoding of the Previous-Trial Face Information

To test whether the previous-trial face information was present during the perception of the current-trial face image, I attempted to decode face identity and facial expression presented in the previous trial based on ERP signals for the current trial. To that end, I organized the data with respect to the previous-trial face information (collapsing across the face information for the current trial). In the previous-trial facial expression decoding analysis, for example, a given trial was labeled as happy if that trial was preceded by a happy face in the previous trial irrespective of true facial expression for that trial. Likewise, in the previous-trial face identity decoding analysis, a given trial was labeled as identity 1 if that trial was preceded by identity 1 in the previous trial irrespective of true face identity for that trial. The first trial was necessarily excluded because previous trial was undefined for the first trial. Note that the face image in the next trial was always in different face identity and facial expression from the previous-trial face image. Thus, the above chance decoding from this analysis cannot be attributed to the stimulus repetition. All other aspects of decoding procedure were identical to the main decoding analysis procedure.

### Code and Data Availability

Both the data and the Matlab analysis scripts are available upon request from G.B.

## Results

### Behavioral Performance

Figure 1c summarizes behavioral performance. The mean accuracy was above chance (= 0.25) for both face identity and facial expression, indicating that participants were actively engaged in the task. Behavioral performance did not differ significantly between face identity and facial expression (t(21) = 0.733, *P* = 0.472, two-tailed). The corresponding Bayes factor (Rouder et al. 2009) (using the default JZS scaling factor of 0.707) showed that the data were 3.23 times more likely to arise from the null hypothesis. These results confirm that participant’s memory for face identity and facial expression was approximately equally accurate.

### Scalp Distributions

The decoding analysis relies on differences in the single-participant scalp distributions of ERP signals for the different face identities and facial expressions. Figure 1d,e shows the grand average scalp maps for each face identity and facial expressions for the duration of perception (averaged across the initial 500 ms from the stimulus onset) and working memory maintenance (averaged across the 1000 ms delay interval). During the perceptual period, the ERP maps show a positive voltage over posterior-occipital scalp sites and a negative voltage over central-frontal scalp sites for both face identity and facial expression. However, during working memory maintenance, the maps show a more positive voltage over centroparietal scalp sites than frontal electrode sites. Although these averaged scalp maps do not show the actual scalp patterns used for decoding (because the decoding analyses were done independently at each time point and separately for each participant), the overall differences in the spatial patterns of ERPs for perception and working memory maintenance period demonstrate that the neural representation of face information is not stable over time. This will be more systematically investigated in the temporal generalization analysis.

### Decoding Face Identity and Facial Expression

**Figure 2:**
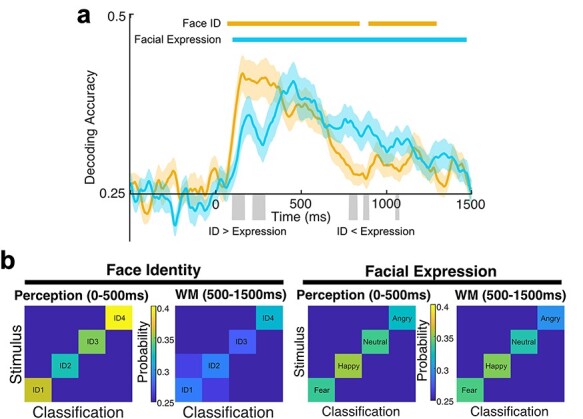
Decoding accuracy and confusion matrices. **a** Time course of mean decoding accuracy for face ID and facial expression (n = 22). Time zero indicates the onset of the stimulus face. Chance-level performance (0.25 = 1/4) is indicated by the abscissa. The colored horizontal lines indicate clusters of time points in which the decoding was significantly different from chance after correction for multiple comparisons. The gray areas indicate clusters of time points in which the decoding was significantly different between face ID and facial expression after correction for multiple comparisons. The light shading indicates ±1 SEM. **b** Confusion matrices for face ID decoding and facial expression decoding for perception (0–500 ms) and working memory (500–1500 ms) periods. Each cell shows the probability that a given ID or expression was classified as a given ID or expression. Cells on the diagonal represent correct classifications.

To track how face-related information is represented during perception and working memory, I applied SVM-based multivariate decoding analysis to the spatial pattern of ERP signals and independently decoded the face identity and the facial expression every 4 ms from −500 ms to 1500 ms relative to the stimulus onset. The decoding accuracy was statistically tested at each time point, using a cluster-based permutation analysis to correct for multiple comparisons (Bae and Luck 2019c). As shown in Figure 2a (colored horizontal lines), decoding accuracy for both face identity and facial expression was significantly above chance (1/4) for most of the time points during the perception and working memory periods (Face Identity: 2 clusters, *P* < 0.001, *P* < 0.001; Facial Expression: 1 cluster, *P* < 0.001). These results replicate previous findings (Nemrodov et al. 2016; Smith and Smith 2019), demonstrating that the spatial pattern of ERP activity contains face-specific information during the perceptual period and extends this finding into the working memory maintenance period.

To confirm that each of the individual face identities and facial expressions was decodable during both perception and working memory maintenance, I performed a confusion matrix analysis by collapsing predicted stimulus labels across all the time points separately during the perception (0–500 ms) and working memory maintenance (500–1500) periods (Fig. 2b). Each of the individual face identities and facial expressions was decodable during both periods, demonstrating that the decoding was not driven by a single distinctive identity or expression. These matrices also confirm that individual facial expressions produced distinctly different neural patterns during both perception and working memory, whereas the neural patterns for individual face identities were much more decodable during perception than during working memory maintenance.

Although ICA was used to correct EEG artifacts associated with eye movements prior to the decoding analysis, it is possible that some residual artifacts might have contributed to the above-chance decoding (Quax et al. 2019). To assess this possibility, I conducted an additional decoding analysis using only the horizontal and vertical EOG channels with radial basis functions (Suykens and Vandewalle 1999). If eye movements were responsible for the decoding, then the signals from these sites should have yielded high decoding accuracy. However, decoding from these sites was much poorer than the main decoding analysis and was not significant for almost all the time points (see Supplementary Material). In addition, I conducted another decoding analysis without trials with extensive eye-movement related artifacts and found the same pattern of the results as in the main analyses (see Supplementary Material). Lastly, to rule out the possibility that the decoding was mainly driven by the different patterns in spatial attention for different types of the stimulus, I conducted the same analysis using the power of alpha-band (8–12 Hz) EEG activity which has known to reflect the shift of spatial attention (Rihs et al. 2007; Foster et al. 2016) and found poor decoding accuracy for both face identity and facial expression for almost all the time points (see Supplementary Material). These results demonstrate that the above-chance decoding in the main analysis was not simply driven by eye-movement artifacts or by differences in spatial attention.

The finding that face identities and facial expressions produced distinctive neural patterns during perception and working memory maintenance does not rule out the hypothesis that integrated representations of the two face dimensions exist in the neural signals. Indeed, the specific combinations of the two face dimensions were also decodable based on the spatial pattern of ERP signals both during perception and working memory maintenance periods (see Supplementary Material). This result demonstrates that spatial pattern of ERP signals contains information about the two independent face dimensions as well as the specific combination of the two face dimensions. In the following analyses, I focus more on the independency of the two face dimensions.

### Independency of Face Identity and Facial Expression

The main decoding analysis demonstrates that the scalp distribution of ERP signals contains at least partially independent information about face identity and facial expression during both perception and working memory maintenance. As a more stringent test of independence, I performed a cross-dimension decoding analysis where a given face feature was decoded while controlling the other feature dimension (Carlson et al. 2014; Grootswagers et al. 2017; Bae and Luck 2018). For identity decoding, the decoder was trained with the data from 3 of the expressions and then tested with the data from the fourth expression. For expression decoding, the decoder was trained with the data from 3 of the identities and then tested with the data from the fourth identity. This requires that the decoder can generalize across expressions when decoding identity and can generalize across identities when decoding expression. This cross-dimension analysis also controls information that is not necessarily related to the dimension of interest. For example, a given facial expression might be decodable due to some potential interaction between the facial expression and other visual features such as hair-style and/or skin tone. This is less likely in the cross-dimension analysis because different face identities were used to train and test the facial expression classifier (see Methods).

**Figure 3:**
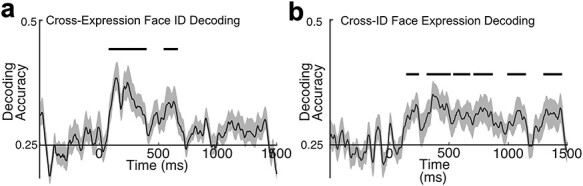
Mean decoding accuracy from cross-dimension decoding analysis for (**a**) face ID and (**b**) facial expression (n = 22). Time zero indicates the onset of a face stimulus. Chance-level performance (0.25 = 1/4) is indicated by the abscissa. The black lines indicate clusters of time points in which the decoding was significantly different from chance after correction for multiple comparisons. The gray shading indicates ±1 SEM.

Although the results from this analysis were noisier due to the lack of iterative random subsampling procedure (see Supplementary Material), the pattern of results from this analysis was consistent with the main recoding analysis. Decoding accuracy for face identity was significantly above chance during the perception period (one cluster, *P* < 0.001) but no significant clusters were observed except for the early working memory maintenance period (one cluster, *P* = 0.025) (Fig. 3a). In contrast, decoding accuracy for facial expression was above chance for the most of time points during perception (2 clusters, *P* = 0.027, *P* < 0.001) and working memory maintenance (4 clusters, *P* = 0.011, *P* = 0.007, *P* = 0.011, *P* = 0.014) (Fig. 3b). These results provide stronger evidence for at least partial independence of face identity and facial expression representations.

### Temporal Generalization of Face Information

**Figure 4:**
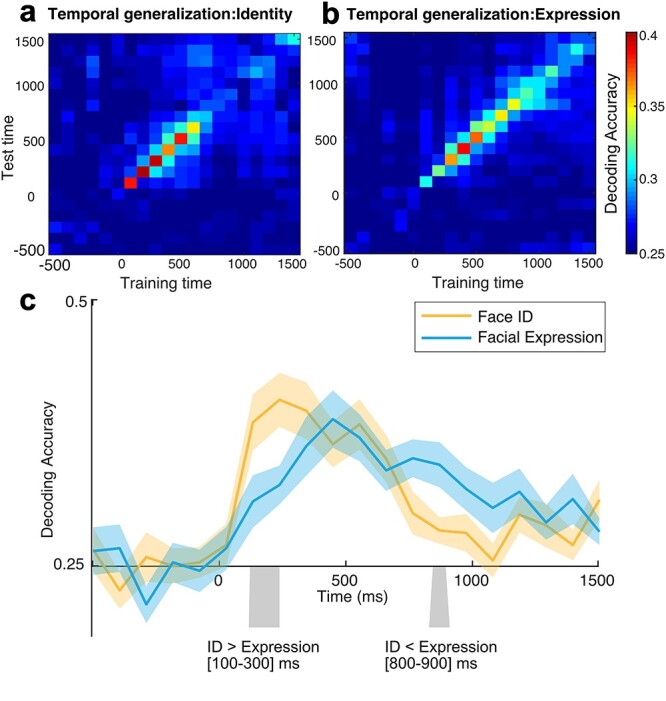
Temporal generalization of decoding for (a) the face ID and (b) the facial expression. The data were averaged for every 100-ms time window prior to the decoding analysis. The color scale represents decoding accuracy (chance = 0.25). c. Time course of mean decoding accuracy for face ID and facial expression when training and testing were done on the same time window (i.e., upper diagonal of panel (a) and (b)) in the time-averaged decoding. The gray areas indicate time windows in which the decoding was significantly different between face ID and facial expression after correction for multiple comparisons. The light shading indicates ±1 SEM.

The scalp patterns of ERP activities for face identity and facial expression were dramatically different between perception and working memory maintenance period, suggesting that the pattern of neural activity for faces dynamically evolve over time (Fig. 1d,e). To test this more systematically, I conducted cross-time decoding analysis (Grootswagers et al. 2017). The logic behind this analysis is that if the pattern of neural signals at one time point persists at different time points, then the classifier trained on one time point should be generalizable to different time points. For the sake of efficiency, the data were averaged within a 100-ms time window prior to the decoding. As can be seen from Figure 4a and b, the decoding was stronger when the classifier was trained and tested on the same time window for both face identity and expression (upper diagonal) and it dropped off away from the diagonal. However, the decoding was still above chance level for nearby time windows, indicating that the neural pattern persists to at least within small time windows (~100 ms). However, the decoding became chance level as the time for training and testing increased, suggesting that the persistent neural activity is limited and that the pattern of neural signals dynamically evolve over time (Wolff et al. 2017).

### Dissociable Temporal Dynamics Between Face Identity and Facial Expression

The face ID decoding was performed collapsed across expressions, and the facial expression decoding was performed collapsed across identities. The above-chance decoding accuracy for each dimension suggests at least some independency in the representation of identity and expression. This independency was also evident in the time course of decodability (Fig. 2a). The decoding of the face identity rose above chance rapidly after the onset of the stimulus and then fell, but remained significantly above chance for some of the time points during the working memory maintenance (see orange lines in Fig. 2a). In contrast, the decoding of facial expression rose more slowly and then fell more slowly, remaining above chance for the most of the time points during the working memory maintenance (see blue lines in Fig. 2a). Indeed, a cluster-based permutation analysis (see Methods) showed that decoding accuracy was significantly greater for face identity than for facial expression during the perception period (2 clusters, *P* < 0.001, *P* < 0.001, two-tailed), but decoding accuracy was significantly greater for facial expression than for face identity during the working memory maintenance period (3 clusters, *P* < 0.001, *P* < 0.001, *P* < 0.001, two-tailed).

The same pattern of results was obtained in the follow-up analyses. In the cross-dimension decoding (Fig. 3), facial expression decoding was above chance for the most of the time points during working memory maintenance, whereas face identity decoding was not. A cluster-based permutation analysis showed a significant cluster of time points at which face identity decoding was greater than facial expression decoding during the perception period (1 cluster, 76–140 ms, *P* < 0.001, two-tailed) and small but significant clusters of time points at which facial expression decoding was greater than face identity decoding during later perception period (1 cluster, 396–420 ms, *P* < 0.001, two-tailed) and at the end of working memory maintenance period (1 cluster, 1436–1452 ms, *P* < 0.001, two-tailed).

This was also evident in the cross-time decoding (Fig. 4c). Facial expression decoding was more reliable than face identity decoding during the working memory maintenance period when the decoder was trained and tested on the same time windows. Comparing decoding accuracy between face identity and facial expression in this analysis showed a significant time windows for better face-identity decoding during perception period (100–300 ms, *P* = 0.007, two-tailed permutation analysis) and a time window for better facial-expression decoding during working memory maintenance period (800–900 ms, *P* = 0.023, two-tailed permutation analysis).

The results from the 3 analyses consistently showed dissociable temporal dynamics of face identity and facial expression. However, it is possible that these results were driven by the 6-Hz low-pass filtering used in the EEG processing prior to the decoding analyses. To rule out this possibility, I conducted additional decoding analysis using a more modest low-pass filtering (~35 Hz) and found the same pattern of results, including the temporal dissociation of face identity and facial expression (see Supplementary Material).

It is intriguing that the information about face identity in the ERP signal declined more rapidly than information about facial expression during working memory. Behavioral analysis showed that the decreased face decoding did not lead to poorer working memory performance for face identity (Fig. 1c). Although these behavioral data do not show that the information about face identity was continuously maintained in working memory, they suggest that the information was accurately maintained somehow in the brain. How was the face identity information maintained during working memory maintenance? One possibility is that the face identity information might be stored in an “activity-silent” manner (Wolff et al. 2017) so that the maintenance of face identity information did not produce decodable neural signals. Although this possibility cannot be directly tested in the context of the present study, I attempted to find evidence for an activity-silent representation of face identity information by asking whether the face identity representation would be reactivated when the next trial begins. This hypothesis is based on previous research showing that orientation representations fade away during working memory maintenance but are then reactivated when the next stimulus appears, indicating that an activity-silent representation must have been present (Bae and Luck 2019b). If face identity information is maintained in an activity-silent manner (consistent with the weaker decoding accuracy for face identity information during the working memory period), then it is possible that this information may be reactivated when the next stimulus appears. However, because the facial expression representations remain decodable through the delay period, there would be no need to reactivate them when the next stimulus appears. This leads to the prediction that the identity of a given face will become decodable again following the appearance of the next face.

**Figure 5:**
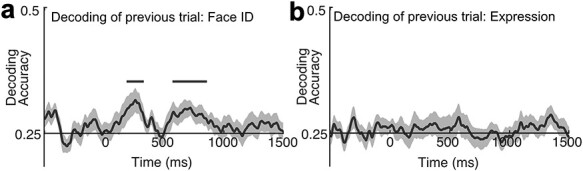
Mean accuracy for (**a**) the decoding of the previous-trial face ID and (**b**) the previous-trial facial expression (n = 22). The black lines indicate clusters of time points in which the decoding was significantly different from chance after correction for multiple comparisons. The gray shading indicates ±1 SEM.

To decode previous-trial face information, I reorganized the data on the basis of the face identity and expression labels from the previous trial (as opposed to the current trial) and attempted to decode the face identity and facial expression. The decoding accuracy for the previous-trial face identity was at chance level before the onset of the current trial stimulus but rose above chance level shortly after the onset of the current-trial stimulus (2 clusters, *P* = 0.021, *P* =.002, one-tailed), suggesting that the previous-trial face identity was reactivated by the current trial stimulus (Fig. 5a). This reactivation of face identity information was not driven by the participants perceiving the same face image in a subsequent trial because stimulus repetition between the consecutive trials was not allowed in the experiment (see Methods). Although it is possible that the chance level decoding during the pre-stimulus baseline period might be due to the baseline correction procedure, the increase of decodability suggests that more information about the previous-trial face identity was available after the onset of the current-trial stimulus. I also conducted the corresponding analysis for facial expression. Results showed that the decoding of the previous-trial facial expression was not significantly greater than chance during the entire time period (Fig. 5b). To further support these results, I conducted a signal-to-noise ratio analysis using the method developed in a previous study (Bae et al. 2020) and found that the signal-to-noise ratio for the previous-trial face identity tended to be greater than the signal-to-noise ratio for the previous-trial facial expression (see Supplementary Material). Although these results provide indirect evidence for activity-silent maintenance of face identity information, they are at least consistent with the hypothesis that different mechanisms were involved in the working memory maintenance of face identity information and facial expression information.

## Discussion

While it is critical to accurately perceive faces for successful social communications, it is also critical to protect the perceived face information from potential interruptions (e.g., occlusions and eye-movements) while the information is maintained in working memory. Although numerous prior studies have shown that face information is efficiently extracted during perception (Crouzet 2010; Martin et al. 2018), it remains unknown how the perceived face information unfolds overtime beyond perception period. The present study investigated the time course of both invariant and changeable aspects of face information, and yielded strong evidence of differential temporal dynamics for face identity and expression during perception and working memory maintenance. Although face identity was more rapidly and accurately decodable than facial expression during perception, facial expression was more decodable during working memory maintenance. These results indicate that identity and expression information are processed at least partially independently.

One intriguing finding in the present study was the rapid decrease of face identity information during the working memory period. The finding of comparable behavioral performance for identity and expression suggests that the face identity information was not simply lost in working memory. Instead, the present results suggest that face identity information was maintained via activity-silent representations (without electrophysiological activity) (Stokes 2015; Wolff et al. 2017). Although the weaker decoding accuracy for face identity during the working memory period could simply indicate that storage of this dimension is not easily detectable from the scalp, other evidence also supported the hypothesis of activity-silent storage. Specifically, although the face identity decoding faded during the delay period, it became decodable again after the onset of the next stimulus. In other words, the face identity apparently shifted into an active, decodable state form when the next stimulus appeared. By contrast, the facial expression remained decodable during the working memory period and was not reactivated when the next stimulus was presented. These different patterns suggest that dissociable mechanisms were used to maintain face identity information and facial expression information. However, the decoding of face identity was significantly greater than chance for some proportion of time points during the late working memory delay (Fig. 2a) and the size of the significant cluster for the difference between the two types of face information was relatively small compared to the size of the working memory maintenance period. Thus, one cannot conclude that face identity information was completely absent in active working memory. Instead, the result suggests that face identity information was maintained in an activity-silent manner on some proportion of trials.

The reactivation of the previous-trial face information also demonstrates that the information in the activity-silent state can be reactivated even if the information is irrelevant to the current-goal of the task (Barbosa et al. 2020). Interestingly, this is not consistent with past studies showing that the reactivation occurs only for a stimulus that might become relevant for future behavior (Rose et al. 2016; Wolff et al. 2017). Investigating the exact cause of this discrepancy is beyond the scope of the present study, but the differences in the task paradigm between the present study and the previous studies suggest that the reactivation observed in the present study might be based on different reactivation mechanisms. Specifically, the previous studies used a retro-cueing paradigm to have participant shift their focus of attention from one working memory representation to another, and found that the initially irrelevant information was reactivated when it becomes relevant. This is clearly different from the task used in the present study in which participants do not need to shift their attention from one representation to another and no explicit demand for the switching was imposed. It would be interesting for future research to investigate whether the reactivation observed in the previous study is driven by the same reactivation mechanisms observed in the present study via a retro-cueing paradigm.

However, the present results are consistent with the previous orientation working memory study that showed the reactivation of the orientation information in the previous trial during perception of the current-trial orientation (Bae and Luck 2019b). This naturally raises the question of why the face information in the previous trial was reactivated. One possible answer would be that the visual system may integrate information over different time points to achieve perceptual stability (Fischer and Whitney 2014). Indeed, a recent study demonstrated that the face representation in the current trial was biased by the face perceived in the previous trial (Liberman et al. 2014), indicating that the previous-trial face information was present during the perception of a new face. Although the present study does not provide behavioral evidence for the serial dependence effect due to the way the face memory was tested, it suggests that the reactivation of previous-trial face information is a potential mechanism for the face serial dependence effect.

However, it is not clear why it is the face identity information, not the facial expression information, that was maintained in an activity-silent manner. One possibility is that the signal-to-noise ratio was lower for the face identity information compared to the facial expression information during the working memory maintenance period. Alternatively, it is also possible that categorical information of face identity was sufficient to perform the task due to the invariant nature of the face identity information, whereas more precise information about facial expression was necessary because it is changeable face information. In case of our daily conversation, it is not necessary to actively hold the identity information in working memory and monitor potential changes in the identity information during the conversation because other’s face identity does not change over the period of the conversation. However, it would be critical to hold the exact facial expression information in mind to notice possible changes in facial expression because the change of facial expression provides important information about other’s ongoing emotional state. It would be an important future research to test whether the more passive maintenance of face identity is an inherent property of face memory or whether this pattern might vary depending on task demands. For example, investigating whether the prioritization of one face dimension over the other leads to differential pattern of reactivation would be important for understanding how attention influences the maintenance of information in working memory (Smith and Smith 2019).

Lastly, one potential limitation of the present study is that the exact source of the decodability is not known. This is a general issue of EEG decoding studies because the source of the signal is generally unknown due to the low spatial resolution of EEG signal and because a decoding algorithm finds any differences that separate one class from the others in a given data. For example, participants in the present study might have used verbal labels to remember the face information and the decoder might have picked up neural signals associated with the verbal labels. Although it is not clear how such strategies would produce the timing pattern observed in the present study, the present study cannot completely rule out the possibility of the labeling strategies. Thus, future research should investigate the exact source of the neural signals associated with perception and working memory maintenance of face information.

## Supplementary Material

Face_Decoding_Bae_SOM_Final_tgaa093

## Footnotes

1 The present study defines working memory “maintenance” period as time points at which the perceived face information has to be maintained in working memory because the face image is no longer available.

## Notes

I thank Steven Luck for useful comments on the experiment design and on the earlier version of the manuscript, and Aaron Simmons for assistance with data collection. *Conflict of Interest*: None declared.
