# Supplementary material for: The Time Course of Face Representations during Perception and Working Memory Maintenance
Source: Cereb Cortex Commun. 2020 Dec 15;2(1):tgaa093. doi: 10.1093/texcom/tgaa093 (PMC8152903; doi:10.1093/texcom/tgaa093)
Supplement: Face_Decoding_Bae_SOM_Final_tgaa093 [file face_decoding_bae_som_final_tgaa093.pdf]

Supplementary material

for

The time course of face representations during perception and working memory maintenance

---

Gi-Yeul Bae

Department of Psychology
Arizona State University
Tempe, AZ, 85287

Address for correspondence:

Gi-Yeul Bae, Ph.D.
Department of Psychology
Arizona State University, Tempe
950 S. McAllister Ave.
Tempe, AZ 85287
(M) 410-491-5540
(E) gbae2@asu.edu

Contents:

1. Decoding integrated representation of face id and facial expression
2. Decoding with EOG channels
3. Decoding without trials with extensive eye-movements
4. Decoding with alpha band (8-12Hz) activity
5. Decoding face id and expression with modest filtering (~35Hz)
6. Decoding face id and expression with different frequency bands (delta, theta, alpha, & beta)
7. Decoding face id and expression without iteration
8. Signal-to-noise ratio analysis for the previous-trial face information

1. Decoding integrated representation of face id and facial expression

The main analysis demonstrated the face identity and facial expression were independently decodable based on the spatial pattern of ERP. In this supplementary analysis, I examined whether the specific combination of face identity and facial expression is also decodable. The decoding method used here was identical to the method used in the main analysis except that the decoder classified the signal into one of 16 classes (4 face ids x 4 facial expressions). This decreased the number of trials used per class compared to the number of trials used in main decoding analysis. For the efficiency of decoding analysis, decoding was conducted every 20-ms time point. As can be seen from Figure S1, decoding was above chance shortly after the onset of the stimulus and continued to be significant almost until the end of the working memory delay interval. This result demonstrates that the spatial pattern of ERP contains information about the integration of face identity and facial expression. Combining this result with the results of independent decoding of face identity and facial expression reported in the main paper suggests that the EEG is a multiplexed signal that concurrently contains different types of information (e.g., one that represent each facial dimension independently and one that represents their combination).

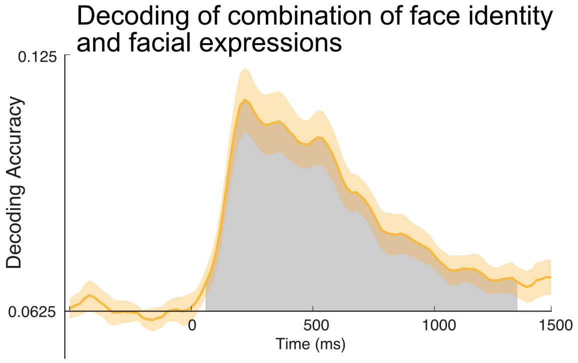

Figure S1. Decoding accuracy of specific combination of Face identity and Facial expression. Chance level is .0625 (= 1/16). Orange and blue shadings represent ±1 SEM. Gray area represents statistically significant clusters of time points.

2. *Decoding with EOG channels*

To test whether the main decoding results were driven by systematic eye movement, I conducted a decoding analysis using HEOG/VEOG channels. If participants moved their eyes systematically depending on face identity and facial expression, then the eye movement should have produced systematic changes in the voltage across the EOG channels. This possibility was tested by decoding face identity and facial expression using EOG channels (Left-EOG, Right-EOG, Upper-EOG, Lower-EOG) without ICA-based artifact correction for eye movement. To compensate the smaller number of channels, I used radial basis function together with ECOC-support vector machine. Results showed poor decoding performance for both face identity and facial expression (Figure S2a,b).

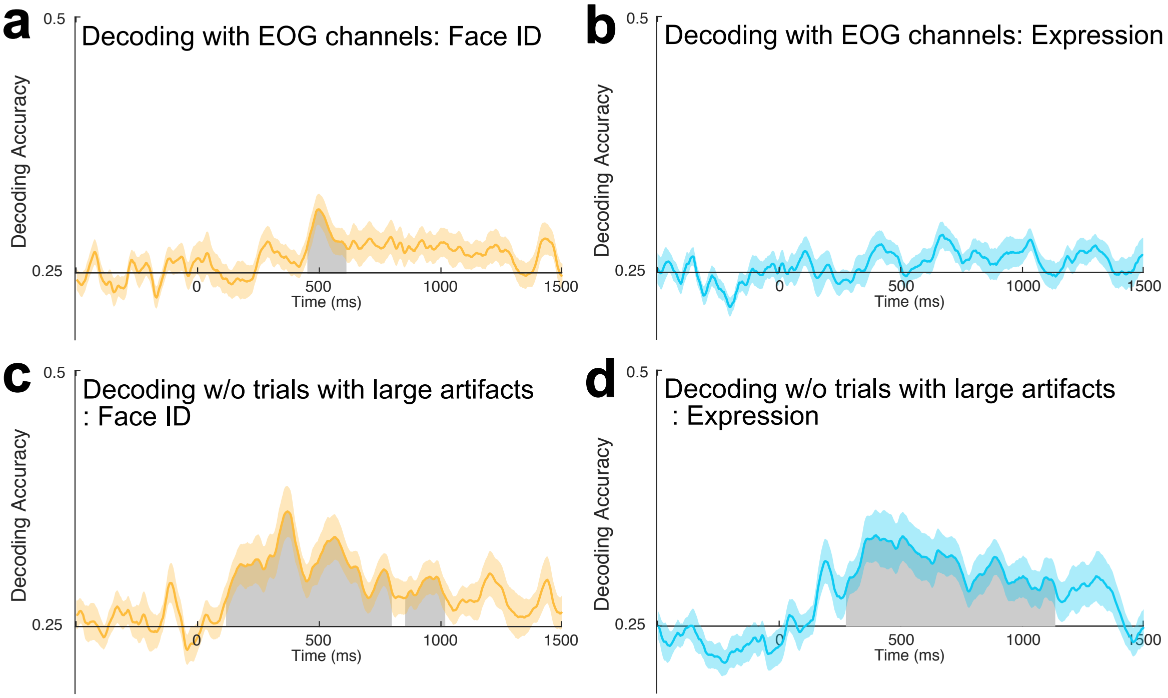

Figure S2. Decoding with HEOG and VEOG channels for (a) Face identity and (b) Facial expression. Decoding after removing trials with large artifacts in EOG channels for (c) Face identity and (d) Facial expression. Orange and blue shadings represent ±1 SEM. Gray area represent statistically significant clusters of time points.

3. *Decoding without trials with extensive eye-movement*

To further ensure that the results from the main decoding analysis was not based on signals related to eye positions, I conducted a set of new decoding analyses by excluding trials that could potentially involve systematic eye movements. To remove trials with potential eye movements, I first computed the mean HEOG (Right EOG – Left EOG) and VEOG (Lower EOG – Upper EOG) over the stimulus presentation period, and converted the voltage (μV) into corresponding units of degrees (°) using normative scaling values for HEOG (16 μV/°) and VEOG (12 μV/°) (Lins et al., 1993). The converted vector represents angle and amplitude of the eye position relative to fixation. I then excluded trials from decoding analysis if the amplitude of the eye position was greater than 0.5° in any directions. This exclusion criterion was even more conservative than the criterion used in the previous orientation decoding study (Bae & Luck, 2018), excluding approximately 56% of the total trials. As a result, the amplitude of the average eye position for a given stimulus in the remaining trials was very small (0.30°, SEM = 0.002°). However, this removal procedure decreased the number of trials for averaging, thus it was expected that the decoding performance from this analysis should be nosier than the main decoding analysis. This removal procedure also produced unequal number of trials across the stimulus class. However, I used the same number of trials across the stimulus class in the decoding (i.e., using the smallest number of trials for all classes). The remaining procedure for the decoding analysis was identical to the main decoding analysis. Despite the removal of extensive number of trials, both face identity and facial expression were decodable during both perception and working memory maintenance (Face ID: 2 clusters, $p<.001$, $p<.001$; Facial expression: 1 cluster, $p <.001$) (Figure S2c and d). Statistical comparison of decoding accuracy between the two face dimensions did not produce significant clusters of time points, presumably

because of decreased signal-to-noise ratio. Thus, I do not make strong conclusions on the temporal aspects of face representations based on this null result. However, the pattern of results was at least consistent with the main results. During the perception period, face identity decoding was above chance for the most of the time points whereas facial expression decoding was above chance only for the second half of the time points. During the working memory maintenance period, face identity decoding showed two discrete clusters of significant time points during the first half (500-1000 ms) of the maintenance period whereas facial expression decoding was continuously above chance beyond the first half of the maintenance period.

4. Decoding with alpha-band activity

To rule out the possibility that the decoding was mainly driven by the different patterns in spatial attention for different types of the stimulus (Foster et al., 2016), I conducted the same analysis on the basis of oscillatory alpha-band (8-12 Hz) activity. The segmented EEG was bandpass filtered at 8-12 Hz and then submitted to a Hilbert transform to compute the total power at each time point. Results showed only a small significant time cluster for face identity (1 cluster, $p = .001$) and facial expression (1 cluster, $p = .011$) (Figure S3). These results demonstrate that the above-chance decoding in the main analysis was not simply driven by differences in the pattern of spatial attention.

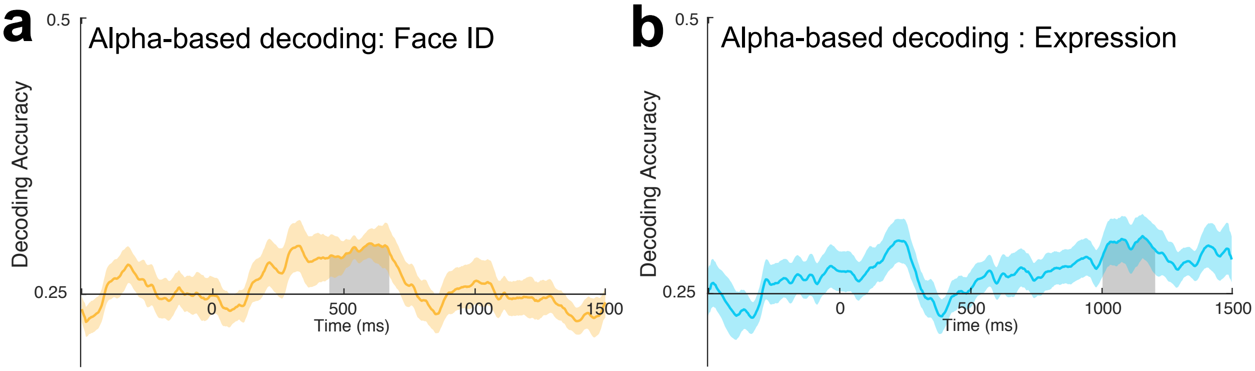

Figure S3. Decoding of Face identity (a) and Facial expression (b) based on the spatial pattern of the power of alpha-band activity (8-12 Hz). Orange and blue shadings represent ±1 SEM. Gray area represents statistically significant clusters of time points.

5. Decoding face id and expression with modest filtering

The temporal dissociation of face identity and facial expression decoding reported in the main text could be driven by the aggressive lowpass filtering (~6HZ) used in the EEG processing. To rule out this possibility, I conducted additional decoding analysis using lowpass filtering at 35 Hz.

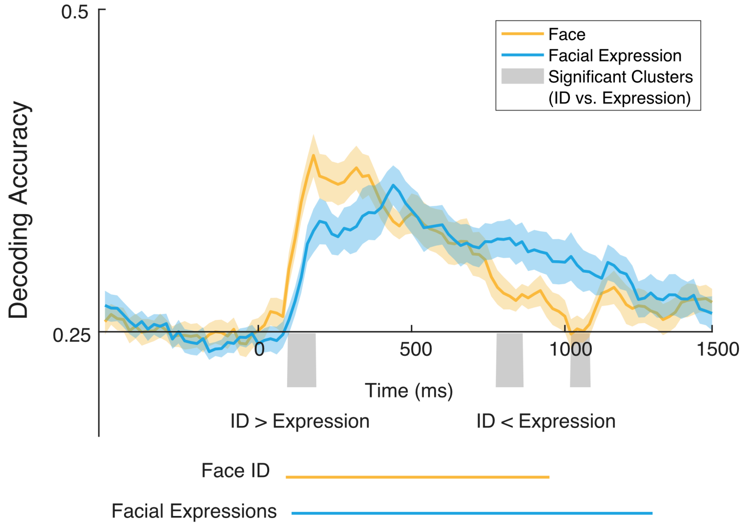

Figure S4. Decoding accuracy for face identity and facial expression when the data was lowpass-filtered at 35Hz. Colored horizontal lines on the bottom indicate clusters of time points in which the decoding was significantly different from chance after correction for multiple comparisons. Gray areas indicate clusters of time points in which the decoding was significantly different between Face ID and Facial expression after correction for multiple comparisons. The light shading indicates ±1 SEM.

Figure S4 represents average accuracy of face identity decoding and facial expression decoding. Face identity decoding was above chance starting from early perception period until around 1000-ms in the delay interval (one cluster, $p <. 001$, one-tailed permutation testing). However, facial expression decoding was above chance from a slightly later time point compared to the face identity decoding but it continued to be significant almost until the end of the delay interval

(1 cluster, p < .001, one-tailed permutation). When I compared the decoding accuracy between face identity and facial expression, I found a cluster of time point in the perception period where face identity decoding was greater than facial expression decoding (1 cluster, p < .001, two-tailed permutation) and two clusters of time points in the working memory maintenance period where facial expression decoding was greater than face identity decoding (2 clusters, p <.001, p <.001, two-tailed permutation). These results are consistent with the main decoding analysis, demonstrating that the main results are not driven by the lowpass filtering used in the EEG processing.

6. Decoding with ERPs from different frequency bands

I explored decoding performance based on ERPs from different frequency bands (Delta: 0.1-4Hz; Theta: 4-7Hz; Alpha: 8-12Hz; Beta: 12-36Hz). Decoding method used in this analysis was identical to the main decoding analysis except that decoding was done every 20 ms (as opposed to 4 ms in the main analysis) for the sake of efficiency. As can be seen from Figure S5, decoding was robust during both perception and working memory maintenance for the ERPs from delta-band (similar frequency band used in the main analysis) whereas the decoding based on other frequency bands was limited to the perception period.

For the decoding with delta-band activity, I compared whether face identity and facial expression exhibit differential temporal dynamics as reported in the main text. I found a significant cluster of time points where face identity was greater than facial expression during perception period (1 cluster, 100-240 ms, p < .001, two-tailed permutation testing) and a significant cluster of time points where facial expression was greater than face identity during

working memory maintenance period (1 cluster, 780-900 ms, $p < .001$, two-tailed permutation testing). These results are consistent with the main results.

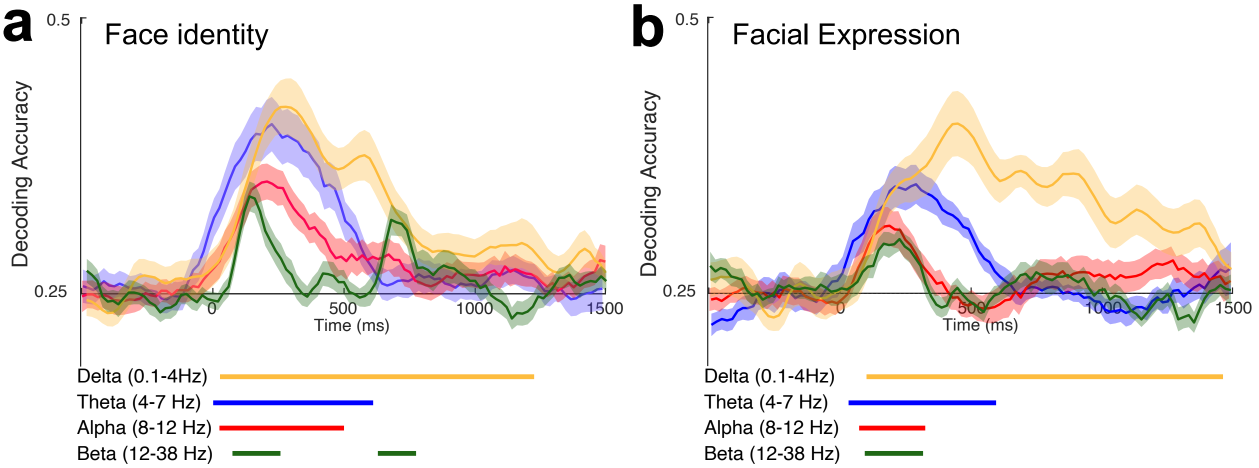

Figure S5. Decoding accuracy for face identity and facial expression with ERPs from different frequency bands. Horizontal lines on the bottom of each panel represents significant clusters of time points. The light shading indicates ±1 SEM.

7. *Decoding without the iterative random subsampling procedure*

I conducted additional decoding analysis without the iteration with random shuffling procedure. As can be seen from Figure S6, the size of significant cluster of time points was smaller and the decoding was less continuous in the decoding without iteration (significant cluster: Face ID: 100-732 ms; Facial Expression: 128-704 ms and 1016-1144 ms) compared to the decoding with multiple iterations (significant cluster: Face ID: 26-828 ms and 900-1280 ms; Facial Expression: 104-1456 ms in the main decoding). Specifically, Face identity decoding without the iteration procedure produced a significant cluster of ~600 ms during the early time period whereas Face identity decoding with the iteration procedure produced a significant cluster of ~800 ms and another significant cluster of ~400 ms. Facial Expression decoding without the iteration procedure produced a significant cluster of ~600 ms and another small cluster of 120 ms whereas

Face identity decoding with the iteration procedure produced a significant cluster of ~1400 ms which covers almost all the time points in the epoch. These results demonstrate that the decoding without the integration with random shuffling procedure can produce nosier results.

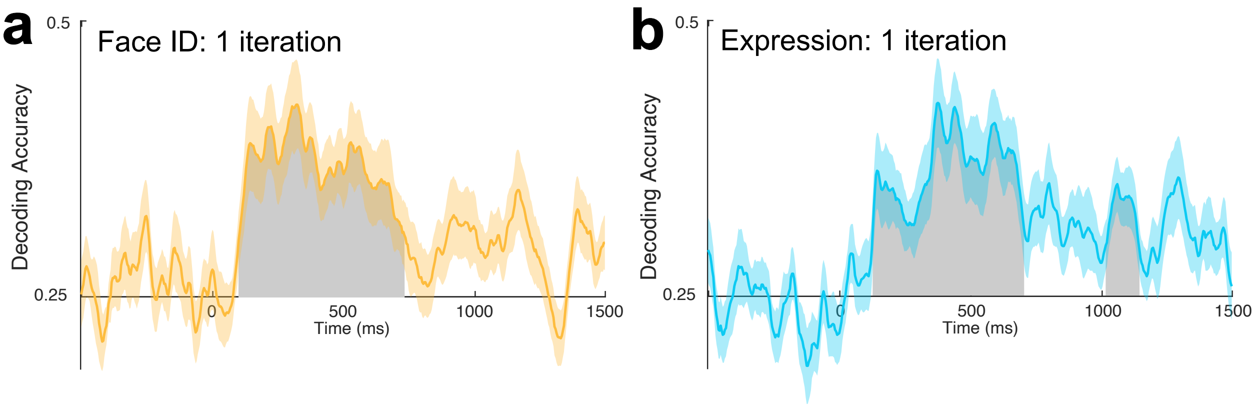

Figure S6. Decoding accuracy for face identity and facial expression without iteration with random shuffling. Gray areas indicate clusters of time points in which the decoding was significantly different between Face ID and Facial expression after correction for multiple comparisons. The light shading indicates ±1 SEM.

8. Signal-to-noise ratio analysis for the previous-trial face information

To further support the reactivation of the previous-trial face identity information, I conducted an analysis to see if the previous-trial face identity indeed exhibited greater signal-to-noise ratio than the previous-trial facial expression using the method developed in a previous study (Bae et al., 2020). The essence of this analysis is similar to the basic idea of ANOVA. Signal was estimated by computing variance in the neural signal created by the stimulus across the electrodes (i.e., interaction between stimulus and the electrodes) and divided that by the corresponding degrees of freedom (RMSsignal). Similarly, noise was estimated by computing variance created by stimulus-independent factors (e.g., variances across different trials) and divided that by the corresponding degrees of freedom (RMSnoise). Finally, signal-to-noise ratio

(RMSratio) was computed by the ratio between RMSsignal and RMSnoise. This was done for the data averaged across 0-1500 ms time points because I did not have a priori hypothesis about the specific time window where this analysis should be applied to. And this analysis was done for each individual participant separately. As can be seen from the figure on the right, face identity exhibited greater signal-to-noise ratio compared to facial expression, consistent with the main decoding results. However, the difference was not statistically significant ($t(21) = 1.54$, $p = .14$) presumably because this analysis is less powerful than the decoding analysis (Bae et al., 2020).

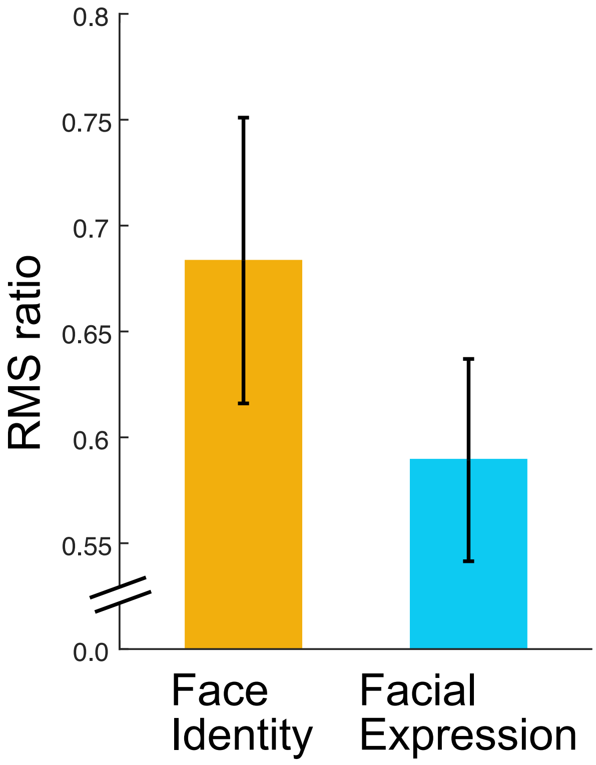

References

Bae, G.-Y., Leonard, C. J., Hahn, B., Gold, J. M., & Luck, S. J. (2020). Assessing the information content of ERP signals in schizophrenia using multivariate decoding methods. *NeuroImage: Clinical*, *25*, 102179. https://doi.org/10.1016/j.nicl.2020.102179

Bae, G.-Y., & Luck, S. J. (2018). Dissociable Decoding of Spatial Attention and Working Memory from EEG Oscillations and Sustained Potentials. *The Journal of Neuroscience*, *38*(2), 409–422. https://doi.org/10.1523/JNEUROSCI.2860-17.2017

Foster, J. J., Sutterer, D. W., Serences, J. T., Vogel, E. K., & Awh, E. (2016). The topography of alpha-band activity tracks the content of spatial working memory. *Journal of Neurophysiology*, *115*(1), 168–177. https://doi.org/10.1152/jn.00860.2015

Lins, O. G., Picton, T. W., Berg, P., & Scherg, M. (1993). Ocular artifacts in EEG and event-related potentials I: Scalp topography. *Brain Topography*, *6*(1), 51–63. https://doi.org/10.1007/BF01234127
